# Antibacterial Effect of Ethanolic Extracts of *Dodonaea viscosa* L. Jacq. and *Mammea americana* L. against Staphylococci Isolated from Skin Lesions

**DOI:** 10.1155/2023/5584412

**Published:** 2023-09-04

**Authors:** Oscar Alberto Pérez-Narváez, Sandra Loruhama Castillo Hernández S, Catalina Leos-Rivas, Raymundo Alejandro Pérez-Hernández, Abelardo Chávez-Montes, Jorge Armando Verduzco-Martínez, Eduardo Sánchez-García

**Affiliations:** ^1^Department of Chemistry, Laboratory of Analytical Chemistry, Universidad Autónoma de Nuevo León, Faculty of Biological Sciences, San Nicolás de los Garza, Nuevo León C.P. 66455, Mexico; ^2^Food Department, Laboratory of Functional Foods, Universidad Autónoma de Nuevo León, San Nicolás de los Garza, Nuevo León C.P. 66455, Mexico; ^3^Department of Cell Biology and Genetics, Faculty of Biological Sciences, Universidad Autónoma de Nuevo León, San Nicolás de los Garza, Nuevo León C.P. 66455, Mexico

## Abstract

**Background:**

The resistance to antibiotics shown by some dermatological pathogenic microorganisms has increased the interest of pharmaceutical and cosmetic industries in developing natural products that possess different biological activities, including antimicrobial effects.

**Methods:**

In the present investigation, the antibacterial activity of ethanolic extracts of *Dodonaea viscosa* aerial part and *Mammea americana* leaves and seed was evaluated against resistant strains of *Staphylococcus* isolated from skin lesions and against *S. aureus* ATCC 25923 (reference strain). Column chromatography (CC) and preparative thin-layer chromatography (PTLC) were used to obtain separate fractions of the seed extract of *M. americana*. We also determined the antimicrobial resistance of the strains against antibiotics using the agar disc diffusion assay. In addition, phytochemical screening was performed by colorimetric standard techniques.

**Results:**

*M. americana* seed extract showed the highest antibacterial activity with MBC from 2.3 *μ*g/mL to 19.5 *μ*g/mL without differences with gentamicin (*p* = 0.998). The isolated strain *S. epidermidis* I showed the highest antimicrobial resistance against the tested antibiotics. PTLC-fractions of *M. americana* seed extract showed MBC from 3.2 *μ*g/mL to 40.7 *μ*g/mL against *S. epidermidis* I and *S. aureus* 25923 (reference), respectively, which suggests a synergistic effect of the secondary metabolites present in the crude ethanolic extract compared to its active PTLC-fractions, where only coumarins and compounds with lactone groups were detected in the phytochemical screening.

**Conclusion:**

*M. americana* seed extract has promising effects that should be considered in further studies as an alternative or adjuvant in treating skin infections caused by staphylococci.

## 1. Introduction

Human skin is the body's most extensive organ; it has an average surface area of 2 m^2^ in adults and a variable chemical constitution. Its functions include protection, permeability, and stabilization of body temperature [1]. Skin diseases affect about 900 million people of all ages [2], and although their mortality rate is relatively low, they are often persistent and challenging to treat [1, 3]. According to the Global Burden of Disease (GBD), an estimated 66,500 deaths yearly are due to bacterial infections in this organ [2]. The inappropriate use of antibiotics has favored the appearance of antibiotic-resistant microorganisms, making it difficult to combat the diseases they cause [4]. Due to this and the possible side effects of conventional treatments [5], in recent years, there has been an increased interest in natural products by both the pharmaceutical and cosmetic industries in the search for new, safe, and more effective treatments [6], among which are the antimicrobial and antioxidant properties of plants [7]. The secondary metabolites, synthesized by plants [8], could provide beneficial effects on skin health [9]; such products have been accepted for generations due to their efficacy, biodegradability, low environmental impact, and the fact that, when used in appropriate doses, they do not generate harmful effects [6]. For these reasons, developing new antimicrobial plant products as auxiliaries in treating skin infections is a growing trend [4]. Antimicrobial properties of *D. viscosa* and *M. americana* have been previously reported. Regarding this, a significant number of reports highlight the biological activities of *M. americana*, such as potential source of natural antioxidants, antimicrobial and even for the treatment of skin diseases [10, 11]). Furthermore, a recent study by Pajaro-Gonzalez et al. [12] reported antibacterial activity at low concentrations of the ethanol extract of the seeds of *M. americana* against *S. aureus* (ATCC and clinical) strains that are sensitive and resistant to methicillin. Regarding *D. viscosa*, several reports of its antibacterial activity can be found in the literature. Getie et al. [13] report that *D. viscosa*, possess antibacterial activity against *S. aureus*, suggesting the potential of this plant to treat bacterial infections of the skin. Similarly, Mothana et al. [14] mention that *D. viscosa*, could be a source for antibacterial drugs against gram-positive bacteria, especially against multiresistant microorganisms. In the present study, we evaluated the antibacterial activity of ethanolic extracts of *D. viscosa* aerial part, and *M. americana* leaves and seed against *Staphylococcus* strains isolated from skin lesions and *S. aureus* ATCC 25923. Besides, the antibacterial activity of fractions obtained by column chromatography and PTLC of *M. americana* seed extract was also evaluated.

## 2. Materials and Methods

### 2.1. Plant Material

*D. viscosa* was collected in Durango, México, and *M. americana* in Quintana Roo, México. The plant material was dried at room temperature in the shade; afterward, the leaves of *D. viscosa* and the aerial part and seeds of *M. americana* were separated. Each plant sample was crushed and pulverized in a manual grain mill. The dried and ground plants were stored in manila paper envelopes and in a fresh and dry place [15].

### 2.2. Preparation of Extracts

For extract preparation, 100 g of dry plant material was weighed on an analytical balance (Nimbus® NBL-214e). Plant material was placed in 1,000 mL Erlenmeyer flasks, to which 500 mL of absolute ethanol (CTR Scientific) was added. Maceration was performed by constant agitation in an orbital shaker (Luzeren® THZ-100) at room temperature for 24 h. Subsequently, the extracts were filtered through filter paper (WHATMAN No.1); the solvent recovered was evaporated using a rotary evaporator (Yamato BM 100) under reduced pressure. Finally, the dried extracts were stored in light-protected vials at 4°C until use [16].

### 2.3. Bacterial Strains and Culture Conditions

Bacterial strains used in this investigation (five strains of *S. epidermidis*, one strain of *S. saprophyticus*, and one strain of *S. aureus*) were obtained from the culture collection of the Analytical Chemistry Laboratory of the Faculty of Biological Sciences. These strains were previously isolated from skin lesions during an independent investigation, carried out from April to June 2018. *S. aureus* ATCC 25923 was used as a reference. All strains were maintained in tubes with Mueller-Hinton Agar slants (DIFCO) at 4°C until use. Before the antimicrobial assay, active cultures were obtained by inoculating a loopful of each strain into 5 mL of Mueller-Hinton Broth (DIFCO) and incubated overnight at 35°C ±2°C [15].

### 2.4. Antibiotic Susceptibility Assay

Bacterial susceptibility was evaluated by the agar disc diffusion assay, according to the Kirby-Bauer technique and following the standards established by the *Clinical and Laboratory Standards Institute* (CLSI). For these, activated cultures were seeded by extension in MH agar plates; subsequently, discs containing antibiotics (MultiBac I.D.) were placed on the agar plate and incubated at 35°C ±2°C for 16 h to 18 h. Finally, the measurement of inhibition zones (mm) was determined and recorded [17]. Antibiotics used were ampicillin (AM) 10 *μ*g, cephalothin (CF) 30 *μ*g, cefoxitin (CFX) 30 *μ*g, ciprofloxacin (CPF) 5 *μ*g, clindamycin (CLM) 30 *μ*g, dicloxacillin (DC) 1 *μ*g, erythromycin (E) 15 *μ*g, gentamicin (GE) 10 *μ*g, penicillin (P) 10 U, sulfamethoxazole+trimethoprim (SXT) 25 *μ*g, tetracycline (T) 30 *μ*g, and vancomycin (V) 30 *μ*g (Multibac Investigación Diagnóstica I.D. Gram positives).

### 2.5. Agar Well Diffusion Assay

The antimicrobial activity of extracts was determined by the agar well diffusion technique. Each strain was spread using a Digralsky loop onto MH agar plates. Then, wells were cut on the agar using an inverted sterile tube (Ø =8 mm in diameter). Subsequently, 100 *μ*L of the extracts were deposited in each well; ethanol was evaluated alone as a control. Plates were incubated overnight at 35°C ±2°C, and after the incubation period, inhibition zones were recorded. This assay was performed three times in triplicate [18].

### 2.6. Minimum Inhibitory Concentration (MIC) and Minimum Bactericidal Concentration (MBC)

The microplate dilution technique was used for MIC determination. For this, different concentrations of the extracts were evaluated in MH broth inoculated with 1% of the bacterial strain [19]. Plates were incubated for 18 h at 35°C ±2°C. The MIC value was determined as the concentration of the extract resulting in complete inhibition of visible growth of the microorganisms evaluated. For MBC, an aliquot of 25 *μ*L was taken from each well without visible growth. Subsequently, drops were dispensed onto an MH agar plate [20]. Plates were incubated under previously established conditions. MBC was defined as the concentration of extracts that eliminated 99.9% of the microorganisms evaluated [21].

### 2.7. Phytochemical Screening

Standard phytochemical tests (Supplementary materials (available here)) to detect metabolites such as carbohydrates, sterols, triterpenes, sesquiterpenes, lactones, tannins, alkaloids, flavonoids, coumarins, quinones, and saponins were performed following standard procedures [22].

### 2.8. Chromatographic Separation

The extract with the highest antibacterial activity was fractionated by column chromatography (CC) using silica gel 60 Ä (Merck®). The extract was eluted with 1 L of the solvent system CHCl_3_-MeOH in different proportions. Fifty-mL portions were collected in 250 mL beakers, and the obtained fractions were concentrated by reduced pressure and resuspended in ethanol (EtOH). The purity of fractions was evaluated using thin-layer chromatography (TLC) [23].

### 2.9. Detection and Semipurification of Antimicrobial Compounds

The bioautography technique was used to detect the active compounds of the fractions obtained by CC. Developed TLC plates were placed face down inside the MH agar plates with the microorganism of interest previously seeded by extension. Plates were incubated at 35°C ±2°C for 24 h to enable the compounds' diffusion. Subsequently, fractions with antimicrobial activity were evidenced by the presence of inhibition zones on the agar surface, corresponding to the antimicrobial compound. Retention factors (Rf) were recorded for further analysis. Finally, active compounds were obtained using preparative thin-layer chromatography (PTLC) by scarping plates eluted as bioautography and according to the RF obtained in the bioautography technique [24, 25].

### 2.10. Statistical Analysis

Data were reported as mean ± SD and were analyzed by ANOVA with Tukey and Dunnett's test. It has statistical significance *p* ≤ 0.05.

## 3. Results and Discussion

### 3.1. Preliminary Inhibitory Effect of Ethanolic Extracts

The antibacterial properties of plant extracts have been demonstrated in a wide variety of studies. [7, 26, 27]. The present investigation observed such activity in the ethanolic extracts of *M. americana* and *D. viscosa.* Both extracts showed inhibition zones (IZ) from 12.5 mm to 30.5 mm against the *Staphylococcus* strains. The activity of the *M. americana* seed extract is outstanding since it produced the highest inhibition effect, ranging from 21.3 mm (*S. aureus* ATCC 25923) to 30.5 mm (*S. epidermidis* I) and because no significant differences were observed with the positive control (gentamycin). *M. americana* aerial part extract was less active showing inhibition zones from 15.2 mm (*S, saprophyticus*) to 22.5 mm (*S. aureus*) (Table 1). Similar results were reported by Poojary et al. [28] using different extracts of *M. suriga* root bark against *S. aureus* ATCC 25923 (IZ = 28 mm-33 mm), attributing this effect to the alkaloids and flavonoids found in the extracts. Likewise, the inhibition zones generated by our *M. americana* seed extract were similar to the inhibition zones of two hydroxycoumarin isolated from *M. africana* named Mammea B/BB, and Mammea B/BA which showed inhibition zones of 15 mm and 18 mm, respectively, against *S. aureus* ATCC 6538 [29].

Regarding the *D. viscosa* leaf extract against *Staphylococcus* strains, the inhibition zones showed ranges from 12.5 mm (*S. saprophyticus*) to 18.8 mm (*S. aureus*), and significant differences were found with the extracts of *M. americana* as well as with the positive control (Table 1). However, these results showed superior activity to those reported by Getie et al. [30]. They reported that the methanolic extract of *D. viscosa* showed inhibition zones of 8 mm to 9 mm against *S. aureus* ATCC 29213. Al-Haj et al. [31] reported inhibition zones of 16.3 mm against S*. aureus*, similar to those obtained in this investigation. Mothana et al. [32] reported the effect of aqueous and methanolic extracts of *D. viscosa* against *S. aureus* ATCC 6538, showing inhibition zones of 14 mm and 15 mm, respectively. Furthermore, methanolic extract was effective against multidrug-resistant *S. aureus* from northern Germany (IZ = 12 mm) and against multidrug-resistant *S. epidermidis* 847 (IZ = 12 mm). Likewise, Monreal [33] reported that the 80% ethanolic extract of *D. viscosa* presented inhibition zones of 10-20 mm against *S. aureus* INDRE-LIBM-01001.

### 3.2. Minimum Inhibitory Concentration (MIC) and Minimum Bactericidal Concentration (MBC)

The MIC values obtained in this work range from 1.7 *μ*g/mL to 1,800 *μ*g/mL, depending on the extract and the strain tested (Table 2). *M. americana* seed extract showed the lowest MIC against the strains of *S. epidermidis* and *S. saprophyticus* with values from 1.7 *μ*g/mL to 2.9 *μ*g/mL. Moreover, the MIC obtained for *S. aureus* (isolated from skin) was 8.8 *μ*g/mL and 17.0 *μ*g/mL against *S. aureus* ATCC 25923. Similarly, low MICs were reported by Pajaro-Gonzalez et al. [12], with MIC_90_ values between 2 *μ*g/mL and 4 *μ*g/mL of the ethanol extract of the seeds of *M. americana* against *S. aureus* strains. However, all the results mentioned above are lower than those obtained by Yasunaka et al. [34] who reported 64 *μ*g/mL of methanolic extracts of the seed of *M. americana* against methicillin-resistant *S. aureus* strains.

*D. viscosa* extract showed higher MIC values from 200 *μ*g/mL to 960 *μ*g/mL, according to the tested strain. Similarly, Verotta et al. [35] reported ranges from 125 *μ*g/mL to 1,000 *μ*g/mL against *S. aureus* ATCC 25923. However, our results are lower than those obtained by Al-Haj et al. [31], who obtained a MIC of 20,000 *μ*g/mL with the methanolic extract of *D. viscosa* against *S. aureus*.

The results of MBC (Table 2) show that *M. americana* seed extract presented the lowest values, ranging from 2.3 *μ*g/mL (*S. epidermidis* IV strain) to 19.5 *μ*g/mL (*S. aureus* ATCC 25923). The MBC reported in this research are lower than those found in the literature. Manjulatha [36] mentioned an MBC ranging from 125 *μ*g/mL to 500 *μ*g/mL using the ethanolic seed extracts of *M. americana* against *Streptococcus mutans* ATCC 25175 (gram-positive bacteria). Similarly, Pajaro-Gonzalez et al. [12] report MBC higher than 64 *μ*g/mL (>64 *μ*g/mL) establishing in their investigation a bacteriostatic effect of the extract. Interestingly, the ethanolic extract of the aerial part of *M. americana* presented greater activity against *S. epidermidis* strains (91 *μ*g/mL to 540 *μ*g/mL) followed by the MBC of isolated and ATCC strains of S. aureus (625 *μ*g/mL and 650 *μ*g/mL, respectively) and finally by S. saprophyticus (1750 *μ*g/mL). On the other hand, we consider that the *D. viscosa* extract was the least active against staph strains, since four strains presented CMB greater than 1000 *μ*g/mL and two strains are above 700 *μ*g/mL. However, Herrera et al. [37] report MBCs ranging from 20,000 *μ*g/mL to 40,000 *μ*g/mL against *S. aureus* strains, using 80% ethanol and diethyl ether extracts of *D. viscosa* leaves, results that are higher than those reported in this investigation.

### 3.3. Antimicrobial Determination of Isolated PTLC Fractions

Phytochemicals can be antimicrobial when MIC values range from 100 *μ*g/mL to 1,000 *μ*g/mL [38]. Our results show that secondary metabolites are present in the ethanolic seed extract and the isolated PTLC-fractions of *M. americana* are promising. Two isolated PTLC-fractions showed antimicrobial activity and presented MIC values of 1.8 *μ*g/mL (PTLC fraction II) and 5.6 *μ*g/mL (PTLC fraction I) against *S. epidermidis* I strain. MIC values for *S. aureus* ATCC 25923 were 7.7 *μ*g/mL (PTLC fraction II) and 36.1 *μ*g/mL (PTLC fraction I), it was determined that the fraction with the highest antimicrobial activity is fraction II (Table 3). *S. epidermidis* I strain and *S. aureus* ATCC 25923 were selected for antimicrobial determination of PTLC fractions because they are the lowest and highest antibacterial sensitivity strains (Table 4). Interestingly, the PTLC-fraction II eluted using a mixture of CHCl_3_:MeOH (9.5 : 0.5) presented a similar MIC to the *M. americana* ethanolic seed extract against the same strain of *S. epidermidis* I, suggesting this effect is due to the coumarins since these compounds were only detected with the Baljet test for lactones in the phytochemical screening (Table 5). More than 1,800 natural coumarins are known, and many have been evidenced to possess high biological activity [39]. In this sense, our MIC results for the PTLC-fractions of the *M. americana* seed extract may be comparable with MICs reported by Verotta et al. [40], with *Mesua ferrea* extract, mesuol compound, and coumarin mixtures against multidrug-resistant *S. epidermidis* 3112. Liu et al. [40] reported MICs of 2 *μ*g/mL against one strain of *S. epidermidis* and 4 *μ*g/mL against two strains of *S.* aureus, using the extract of *M. ferrea.* It should be mentioned that the MICs reported for the mesuol compound were the same than those obtained with the crude extract (2 *μ*g/mL and 4 *μ*g/mL), which is comparable with the results obtained in this investigation with PTLC fraction II and the ethanolic extract of *M. americana*. Finally, the extract of *M. ferrea* and the mesuol compound did not show activity against *S. saprophyticus* strains.

In addition, MIC for PTLC fraction II (7.7 *μ*g/mL) against *S. aureus* ATCC 25923 are comparable with the results reported by Yasunaka et al. [34]; they reported MICs values from 1 *μ*g/mL to 8 *μ*g/mL using two flavonoids isolated from the leaf of *Calophyllum brasiliense* and the fruit skin of *M. americana* named Mammea A/BA and Mammea A/AA, against methicillin-resistant (MRSA) and methicillin-sensitive (MSSA) strains of *S. aureus*. Besides this, El-Seedi [41] determined the MIC of the isolated coumarins asphodelin A 4′-O-*β*-D-glucoside and its aglycone asphodelin A isolated from *Asphodelus microcarpus* and reported 128 *μ*g/mL and 16 *μ*g/mL, respectively, against *S. aureus* IAM1011. It is essential to mention that synthetic coumarin compounds have shown relevant results, such as those observed by Simões et al. [39], with seventy coumarin compounds from antibiotic novobiocin linked to a pyrazole ring against *S. aureus* ATCC 12600; the MIC obtained using these synthetic compounds ranged from 0.5 *μ*g/mL to 128 *μ*g/mL. Those results can be compared with the obtained with PTLC-fraction I (MIC = 36.1 *μ*g/mL) and PTLC-fraction II (MIC = 7.7 *μ*g/mL) against *S. aureus* ATCC 25923 (Table 3). MICs and MBCs of the PTLC-fractions suggest a synergistic effect from the *M. americana* seed extract; the PTLC-fraction II, on the other hand, required almost half the concentration to inhibit *S. aureus* ATCC 25923, suggesting these metabolites possess a more significant antibacterial effect.

### 3.4. Phytochemical Screening

There is a wide variety of polyphenols with antimicrobial properties [27]. The antibacterial effect of our extracts can be due to the presence of different phenolic compounds whose biological activities are related to their molecular structures containing hydroxyl groups, and phenolic rings bind to proteins and bacterial membranes to form complexes that inhibit bacterial growth [19]. Flavonoids, flavones, and flavonols are phenolic compounds synthesized by plants in response to microbial infections and are generally effective *in vitro* against a wide range of microorganisms [18]. In our study, these metabolites were detected by standard phytochemical screening (Table 5) and could be the cause of the effective antibacterial activity in the *M. americana* and *D. viscosa* extracts. The inhibitory effect of these compounds has been related to the perturbation of membrane permeability and the inhibition of enzymes such as ATPase and phospholipase A2 [42]. Other secondary metabolites found in the extracts were tannins, which are abundant in plants, and their antimicrobial effect against bacteria and fungi has been demonstrated [43]. On the other hand, alkaloids were not detected with Dragendorff's reagent, which could indicate antimicrobial activities are mainly by phenolic compounds [19]. Saponins were detectable in all the extracts except in *M. americana* seed; these metabolites can cause membrane rupture, and their hemolytic activity is known [44]. Likewise, coumarins were also revealed; these phenolic substances are benzene fused to *α*-pyrone rings, many of which possess antimicrobial properties. In the present investigation, we believe coumarins are the principal cause of antibacterial activity in *M. americana* extracts because >120 coumarins have been identified in *Mammea* species with insecticidal, antioxidant, anti-HIV, anticancer, antifungal, antibacterial, antimicrobial, and antibiotic effects [34, 40, 45, 46].

### 3.5. Antimicrobial-Resistant Profile

Antibiotic resistance has increased in recent years due to the inappropriate use of these drugs and is a severe health problem worldwide [17]. In our study, we observed penicillin and ampicillin insensitivity in isolated strains. It is worth mentioning that the *S. epidermidis* I strain showed the lowest sensitivity against the gram-positive antimicrobials (Table 4). Likewise, *S. epidermidis* I, *S. epidermidis* IV, *and S. epidermidis* V isolated strains showed multiresistance by presenting insensitivity to four antibiotics [47]. Velásquez et al. [48] determined the antimicrobial profile of 101 isolates of the *Staphylococcus* genus and observed strains that were resistant to 9 antibiotics. Furthermore, coagulase-negative staphylococci showed resistance to penicillin, ampicillin, erythromycin, tetracycline, kanamycin, and clindamycin. In our work, isolated *S. aureus* showed intermediate insensitivity to dicloxacillin, whereas the *S. epidermidis* from our isolated coagulase-negative staphylococci strain was insensitive to gentamicin. Velásquez et al. [48] observed sulfamethoxazole-trimethoprim insensitivity in one strain of *S. aureus,* whereas coagulase-negative staphylococci did not show resistance; in contrast, we observed resistance in *S. epidermidis* I, similarly with the work of Liu et al. [40], who reported insensitivity in a strain of *S. epidermidis*, as well as to erythromycin and gentamicin. Regarding ciprofloxacin, the strain *S. epidermidis* I showed intermediate resistance, comparable to the one reported by Silva et al. [47]; they observed high susceptibility in coagulase-negative staphylococci isolated from infected chronic wounds. Likewise, we observed similarities in sensitivity to tetracycline except in *S. epidermidis* I. This resistance is probably a consequence of inappropriate antibiotic use, such as erythromycin, which is widely used to treat staphylococcal infection [49]. We observed resistance to clindamycin in strains that also showed insensitivity to erythromycin, so cross-resistance should be considered. Isolated strains in this work were sensitive to vancomycin, which agrees with Peixoto et al. [50], who mention that vancomycin has become the first-line therapy for most infections caused by methicillin-resistant staphylococci. It is well known that resistance to antibiotics by pathogenic microorganisms hinders the treatment of diseases [4]. In the case of infected skin wounds, healing is difficult [47], which may result in other inconveniences, such as increased treatment costs, hospitalization, and side effects [5].

## 4. Conclusion

Ethanolic extracts of *D. viscosa* leaf and *M. americana* aerial part and seed showed good antibacterial activity against staphylococci isolated from skin lesions. The *M. americana* seed extract was the most outstanding due to its low MIC/MBC, comparable with the gentamicin effect. The bioactive PTLC-fractions from the seed extract against the most antimicrobial-resistant strain, *S. epidermidis* I and *S. aureus* ATCC 25923, suggest a synergistic effect of the extract from which they were obtained. Currently, the use of medicinal plants for the care and treatment of skin infections is of great interest, and therefore, the search for alternatives to face such resistance is relevant.

## Figures and Tables

**Table 1:** Inhibition zones (mm) of the ethanolic extracts.

Strains	Ethanolic extract			Antibiotic
	*M. americana* seed extract^c^	*M. americana* aerial part extract^b^	*D. viscosa* leaf extract^a^	Positive control, Gentamicin^c^
S. *saprophyticus*	28.9 ± 0.4^∗^	15.2 ± 0.0	12.5 ± 8.2	30.0 ± 0.0
*S. epidermidis* I	30.5 ± 0.3	16.8 ± 8.9	14.1 ± 1.29	30.0 ± 1.93
*S. epidermidis* II	30.4 ± 1.0	16.3 ± 6.3	13.6 ± 2.88	31.3 ± 0.02
*S. epidermidis* III	28.9 ± 0.3	16.2 ± 1.9	14.7 ± 8.9	28.3 ± 0.02
*S. epidermidis* IV	29.4 ± 0.3	16.2 ± 3.5	14.4 ± 5.7	27.7 ± 00
*S. epidermidis* V	26.9 ± 0.3	16.5 ± 2.2	14.6 ± 1.10	29.7 ± 0.02
*S. aureus*	28.8 ± 1.0	22.5 ± 1.16	18.8 ± 1.34	21.0 ± 0.03
*S. aureus* ^25923^	21.3 ± 1.1	16.7 ± 7.6	16.0 ± 4.11	24.7 ± 0.04

^∗^Mean ± SD (3×) of inhibition zone by well diffusion on MH agar. Diameter (*Ø* = 8 mm) included. ^a,b,c,d^ANOVA-Tukey and Dunnet, *p* ≤ 0.05.

**Table 2:** MIC and MBC (*μ*g/mL) of the ethanolic extracts against strains of staphylococci.

Strains	Ethanolic extract						Antibiotic	
	*M. americana* seed extract^c^		*M. americana* aerial part extract^b^		*D. viscosa* leaf extract^a^		Positive control, Gentamicin^c^	
	MIC	MBC	MIC	MBC	MIC	MBC	MIC	MBC
Ss	2.9 ± 0.4^∗^	3.9 ± 0.4	1000 ± 0.0	1750 ± 267	356 ± 82	450 ± 93	<0.05 ± 0	<0.05 ± 0
Se I	1.8 ± 0.3	2.5 ± 0.5	440 ± 89	540 ± 89	417 ± 129	833 ± 258	21.5 ± 1.93	22.75 ± 1.75
Se II	8.8 ± 1.0	10.0 ± 0.0	300 ± 63	350 ± 63	822 ± 288	1478 ± 632	0.09 ± 0.02	0.14 ± 0.03
Se III	1.8 ± 0.3	2.6 ± 0.6	243 ± 19	293 ± 19	960 ± 89	1800 ± 447	0.06 ± 0.02	0.10 ± 0.03
Se IV	1.7 ± 0.3	2.3 ± 0.6	81 ± 35	91 ± 39	200 ± 57	345 ± 166	0.05 ± 00	0.1 ± 0.00
Se V	1.7 ± 0.3	2.4 ± 0.5	150 ± 22	194 ± 30	920 ± 110	1120 ± 110	0.11 ± 0.02	0.16 ± 0.02
Sa	8.8 ± 1.0	10.0 ± 0.0	313 ± 116	625 ± 231	375 ± 134	750 ± 267	0.23 ± 0.03	0.35 ± 0.05
Sa^25923^	17.0 ± 1.1	19.5 ± 0.9	550 ± 76	650 ± 76	700 ± 411	1100 ± 548	0.1 ± 0.04	0.15 ± 0.06

^∗^Media ± SD of 3 experiments. MIC: minimum inhibitory concentration; MBC: minimum bactericidal concentration. ^a,b,c^ANOVA-Tukey and Dunnet, *p* ≤ 0.05. Mean initial and final inoculation: 2.3 × 10^8^ and 5.8 × 10^10^ CFU/mL, respectively. Ss: *S. saprophyticus*; Se: *S. epidermidis*; Sa: *S. aureus*; Sa^25923^: *S. aureus* ATCC 25923.

**Table 3:** MIC and MBC of subfractions from the *M. americana* seed extract.

Strain	Bioactive PTLC fractions			
	PTLC fraction I		PTLC fraction II	
	MIC	CMB	MIC	CMB
*S. epidermidis* I	5.6 ± 1.7	12.8 ± 1.1	1.8 ± 0.4	3.2 ± 0.8
*S. aureus* ^25923^	36.1 ± 0.9	40.7 ± 1.9	7.7 ± 0.8	10.7 ± 1.6

^∗^Mean ± SD (*μ*g/mL) of 3 experiments (3×). MIC: minimum inhibitory concentration; MBC: minimum bactericidal concentration. Mean initial and final inoculation: 4 × 10^7^ and 3 × 10^10^ CFU m^-1,^ respectively.

**Table 4:** Antimicrobial sensitivity profile of *Staphylococcus* strains.

Antibiotic	Strains							
	*S. saprophyticus*	*S. epidermidis*					*S. aureus*	*S. aureus* 25923
		I	II	III	IV	V		
Ampicillin	R	R	R	R	R	R	R	S
Cephalothin	S	S	S	S	S	S	S	S
Cefotaxime	S	S	S	S	S	S	S	S
Ciprofloxacin	S	I	S	S	S	S	S	S
Clindamycin	S	S	S	I	R	R	S	S
Dicloxacillin	S	R	S	S	S	S	I	S
Erythromycin	S	R	S	S	R	R	S	S
Gentamicin	S	R	S	S	S	S	S	S
Penicillin	R	R	R	R	R	R	R	R
Sulfamethoxazole/Trimethoprim	S	R	S	S	S	S	S	S
Tetracycline	S	R	S	S	S	S	S	S
Vancomycin	S	S	S	S	S	S	S	S

^∗^Antimicrobial sensitivity values represented by R: resistance; I: intermediate; S: sensitive.

**Table 5:** Phytochemical screening of extracts and active subfractions.

Phytochemical test	Extract			PTLC fractions	
	*M. americana* seeds	*M. americana* aerial parts	*D. viscosa* leaf	Rf 0.33	Rf 0.86
Liebermann-Burchard	Triterpene	Triterpene	Sterol	—	—
Sesquiterpene Lactones	+	+	—	+	+
Carbohydrates	+	+	+	—	—
Tannins	+	+	+	—	—
Alkaloids	—	—	—	—	—
Flavonoids	+	++	++	—	—
Coumarins	+	+	+	+	+
Quinones	+	+	+	—	—
Saponins	—	+	+	—	—

+: present; -: not present.

## Data Availability

The data used to support the findings of this study are available from the corresponding author upon request.

## Supplementary Materials

Supplementary Materials Standard methods for phytochemical screening.
