# Supplementary material for: Antibacterial Effect of Ethanolic Extracts of Dodonaea viscosa L. Jacq. and Mammea americana L. against Staphylococci Isolated from Skin Lesions
Source: Biomed Res Int. 2023 Sep 4;2023:5584412. doi: 10.1155/2023/5584412 (PMC10495233; doi:10.1155/2023/5584412)
Supplement: Supplementary Materials — Standard methods for phytochemical screening. [file 5584412.f1.docx]

Standard phytochemical tests

The evaluation of chemical composition of *M. americana* and *D. viscosa* extracts were performed suspended 3-5 mg of dried extract in 1 mL of absolute ethanol or distilled water.

**Bayer’s Test for Unsaturation**

Aqueous 1% KMnO_4_ was added dropwise to the extract solution (5 mg/mL in ethanol). Disappearance of the purple color of KMnO_4_ and the appearance of a brown solid precipitate (MnO_2_) was considered positive test [1].

**2.6. Detection of Triterpenes/Steroids (Liebermann-Burchard Reagent)**

Five drops of concentrated sulfuric acid (H_2_SO_4_) and 1 mL of acetic anhydride (C_4_H_6_O_3_) were added to the extract. A color change from violet to blue confirms the presence of steroids [2] and formation of blue-green ring indicated the presence of terpenoids [3].

**2.7. Coumarins**

Three mL of 2 N NaOH was added to 2 mL of aqueous extract. Formation of yellow color indicated the presence of coumarins. Confirmation test was performed by adding 1 mL of 5 N HCl; in this case a colorless solution formed at the upper layer is considered positive [4].

**2.8. Alkaloids**

Ethanolic extracts (20 *μ*L) were applied on TLC plates (Silica Gel 60G, 5 × 10 cm) and eluted using toluene-ethyl acetate-diethylamine (70 : 20 : 10) as solvent system. Alkaloids were detected after spraying Dragendorff’s reagent as orange-brown spots on TLC plates [5].

**2.9. Screening for Sesquiterpene Lactones**

The Baljet reaction (1% Picric acid in 10% sodium hydroxide) was used to detect sesquiterpene lactones in the extracts. Reagents were mixed at a 1 : 1 ratio and added to 1 mL of extracts (2-3 mg). The transformation of the sodium picrate solution’s yellow color to orange-red color confirmed the positive reaction [6].

**2.10. Test for Quinones**

Extracts suspended in ethanol (1 mL) were treated with 1 mL of concentrated sulfuric acid. Formation of red color shows the presence of quinones [9].

**2.11. Carboxyl Group**

The presence of carboxyl groups was evidenced by adding 10 drops of 10% sodium bicarbonate solution; visible bubbles of carbon dioxide were considered a positive reaction [9].

**2.12. Test for Tannins**

Extracts were treated with 1 mL of 5% ferric chloride which was added. The presence of tannins was indicated by the formation of bluish black or greenish black precipitate [8].

**2.13. Shinoda Test**

Few fragments of magnesium metal ribbon (3-4 pieces) were added to 1 mL of ethanolic extract, followed by dropwise addition of concentrated hydrochloric acid. Formation of pink or red color indicated the presence of flavonoids [7].

**2.14. Saponin**

Two mL of distilled water was added to extracts suspended in ethanol and was shaken vigorously. The formation of copious foam layer indicates the presence of saponins [7].

**Carbohydrates**

Five mg of each extract were suspended in 1 mL of distilled water; afterward 2 mL of 0.2% anthrone reagent and 5 drops of concentrated sulfuric acid were added. Dark green color showed the presence of carbohydrates [9].

Literature

[1] A. Ghosh, A. Bandyopadhyay, P. Ghosh, and P. Chatterjee, “Evaluation of antibacterial potentiality of a Cyclopenta Naphthalene tetraol terpenoid isolated from *Curcuma caesia* Roxb,” *Journal of Botanical Science*, vol. 3, no. 1, pp. 27–34, 2013.

[2] M.Boxi, Y. Rajesh,V.R.Kumar,B.Praveen, andK.Mangamma, “Extraction, phytochemical screening and in-vitro valuation of anti-oxidant properties of *Commicarpus chinesis* (aqueous leaf extract),” *International Journal of Pharma and Bio Science*, vol. 1, no. 4, p. 547, 2010.

[3] A. B. Fawehinmi, H. Lawal, S. O. Etatuvie, and F. O. Oyedeji, “Preliminary phytochemical screening and antimicrobial evaluation of four medicinal plants traditionally used in Nigeria for skin infection,” *African Journal of Pure and Applied Chemistry*, vol. 7, no. 2, pp. 44–49, 2013.

[4] S. J. P. Jacob and S. Shenbagaraman, “Evaluation of antioxidant and antimicrobial activities of the selected green leafy vegetables,” *International Journal of PharmTech Research*, vol. 3, no. 1, pp. 148–152, 2011.

[5] H. Wagner and S. Bladt, *Plant Drug Analysis. A Thin Layer Chromatography Atlas*, Springer, New York, NY, USA, 2^nd^ edition, 2001.

[6] M. A. B. Aziz, “Qualitative phytochemical screening and evaluation of anti-inflammatory, analgesic and antipyretic activities of *Microcos paniculata* barks and fruits,” *Journal of Integrative Medicine*, vol. 13, no. 3, pp. 173–184, 2015.

[7]S. K. Rathore, S. Bhatt, S. Dhyani, and A. Jain, “Preliminary phytochemical screening of medicinal plant *Ziziphus mauritiana* Lam fruits,” *International Journal of Current Pahrmaceutical Research*, vol. 4, no. 3, pp. 160–162, 2012.

[8] S. Firdouse and P. Alam, “Phytochemical investigation of extract of *Amorphophallus campanulatus* tubers,” *International Journal of Phytomedicine*, vol. 3, no. 1, pp. 32–35, 2011.

[9] X. A. Dominguez, *M´etodos en Investigaci´on Fitoqu´ımica*, LIMUSA, 1973.
